# The journey to diagnosis for patients with CIDP: results from a real-world international survey

**DOI:** 10.3389/fneur.2025.1748903

**Published:** 2026-02-04

**Authors:** Clémence Arvin-Berod, Febe Brackx, Lucas Van de Veire, Sandra Paci, Yasmin Taylor, Jack Wright, Alejandra Pérez del Real, Jean Philippe Plançon, Richard Sperry, Chelsey Fix, Sarah Dewilde, Yusuf A. Rajabally

**Affiliations:** 1 Argenx, Ghent, Belgium; 2 Services in Health Economics (SHE) BV, Brussels, Belgium; 3 Adelphi Real World, Bollington, United Kingdom; 4 Neuropatías Autoinmunes GBS | CIDP España, Madrid, Spain; 5 European Patient Organisation for Dysimmune and Inflammatory Neuropathies, Le Pouliguen, France; 6 Association Française Contre les Neuropathies Périphériques, Paris, France; 7 GBS | CIDP Foundation International, Conshohocken, PA, United States; 8 Aston Medical School, Aston University, Birmingham, United Kingdom

**Keywords:** chronic inflammatory demyelinating polyradiculoneuropathy, diagnosis, immune-mediated neuropathy, misdiagnosis, time to diagnosis

## Abstract

**Introduction:**

Chronic Inflammatory Demyelinating Polyradiculoneuropathy (CIDP) is a rare autoimmune disorder affecting the peripheral nerves, typically characterized by muscle weakness and sensory deficits. This study seeks to describe CIDP patients’ journey to diagnosis alongside factors influencing misdiagnosis and time to diagnosis.

**Methods:**

We analyzed demographics and diagnostic data reported by neurologists and their patients in Adelphi’s CIDP Disease Specific Programme™. This digital, multinational real-world survey was held in the UK, France, Germany, Italy and Spain between September 2022 and April 2023 (*n* = 542).

**Results:**

Mean (SD) age was 54.0 (12.4) years; 62% of patients were male. Half of the patients reported at least one comorbidity, with anxiety, depression and diabetes being the most common. The mean (SD) number of diagnostic procedures undergone per patient was 19.6 (9.4). An electromyogram and nerve conduction study (98%), complete blood count (82%) and administration of anti-ganglioside antibodies (78%) were carried out most frequently. Most patients had been diagnosed with typical CIDP (68%) and 37% had been misdiagnosed at least once. The most common misdiagnosis was Guillain-Barré syndrome, in 37% of cases. No significant associations were found between misdiagnosis and the variables sex, disease severity at symptom onset, age category, BMI or CIDP subtype. The median (Q1 - Q3) time between symptom onset and diagnosis was 7.0 (3.2–13.0) months. A multiple linear analysis on the log-transform of the time to diagnosis indicated that patients with a long time to diagnosis more often presented with mild symptoms at onset, had variant CIDP and had been misdiagnosed.

**Conclusion:**

Median time to diagnosis for CIDP patients was 7 months; over a third had at some point been misdiagnosed. Mild symptoms, having variant CIDP and having been misdiagnosed were associated with longer time to diagnosis. Further research into the causes of diagnostic delay and the impact of late diagnosis and treatment is needed.

## Introduction

Chronic Inflammatory Demyelinating Polyradiculoneuropathy (CIDP) is a rare immune-mediated disorder with a heterogeneous pathophysiology (1) and presentation. A number of CIDP variants have been identified, including asymmetrical (also known as MADSAM), focal, distal, sensory and motor CIDP. The disease is typically characterized by progressive symmetrical weakness and sensory symptoms in arms and legs which develop over a period of more than 8 weeks. More rapid onset of acute symptoms within 4 weeks has also been observed in up to 13% of patients (2). Traditional first-line treatment options such as intravenous immunoglobulin and corticosteroids aim to alleviate these symptoms and, ideally, induce and maintain long-term remission. However, many patients continue to experience symptoms despite treatment (3, 4), and some do not respond to treatment at all (5). The prevalence of CIDP has been observed to increase with age, is higher in men and ranges between 0.7 to 10.3 cases per 100,000 people (6). This considerable variation in prevalence rates may be explained by the level of neuromuscular expertise involved, as well as the differences between countries or regions (7). The use of different diagnostic criteria has also been implicated herein (8).

The diagnosis of CIDP is typically made using a combination of clinical and electrodiagnostic criteria, potentially supported by ultrasound or magnetic resonance imaging results, cerebrospinal fluid, nerve biopsy or antibody testing (2). However, several factors can complicate the diagnostic process. Firstly, the disease presents heterogeneously, and no reliable biomarkers have yet been identified (9, 10). Secondly, while there is consensus in theory, existing guidelines may be underutilized in practice due to lack of awareness (11) or practical constraints. Thirdly, diagnostic test results have been known to be misinterpreted (12, 13). And finally, mimics – diseases which present similarly to CIDP and its variants – can cause confusion during the diagnostic process (14–16). All of these aspects can prevent patients from receiving an early diagnosis and proper therapies, which are crucial to prevent axonal loss and resulting disability (17–19).

Another consequence of the aforementioned confounding factors is that both over- and underdiagnosis are a common problem (20). Cornblath et al. (21) estimated that up to one-third of patients in the US with a diagnosis of CIDP do not have the disease. In a Dutch sample of 96 patients, 32% had a referral diagnosis of CIDP that was later revised (22). Patients erroneously diagnosed with CIDP may be treated for long periods of time without clear therapeutic effect, potentially posing a substantial cost to not just the patients, but also to the health care system (23). Simultaneously, many patients with CIDP initially receive a different diagnosis: a Dutch and a British study found this to be the case for 20% and 68% of patients in their respective samples (22, 24). An incorrect diagnosis can contribute to a substantial diagnostic delay and ineffective treatment (25). Bunschoten et al. (4) reported that the time between symptom onset and correct CIDP diagnosis was highly variable, with a median duration of 5 months (interquartile range: 2–12 months), and that it took more than a year for a quarter of CIDP patients to receive their diagnosis. According to Chaudhary et al. (24), patients (*n* = 27) that had been referred with a non-CIDP or Guillain-Barré syndrome (GBS) diagnosis experienced a mean delay of 21.3 months (range 2–123 months) before finally being diagnosed with CIDP.

Beyond the studies previously mentioned, there has been little real-world research into CIDP patients’ journey to diagnosis and what patient characteristics might potentially influence it. This study seeks to further elucidate this aspect of CIDP through a secondary analysis of patients’ demographics, comorbidities, symptomatology, disease severity, Inflammatory Neuropathy Cause and Treatment (INCAT) score, experience with (mis)diagnosis, and diagnostic tests, as reported by their physician. We will also look at the association between a number of variables (age category, sex, disease severity at symptom onset, Body Mass Index (BMI) and CIDP type) and misdiagnosis, as well as time to diagnosis.

## Materials and methods

### Study design and data collection

The Adelphi Real World CIDP Disease Specific Programme™ (DSP) is a linked physician and patient cross-sectional survey with elements of retrospective data collection. The survey was conducted in France, Germany, Italy, Spain and the UK between September 2022 and April 2023. Physicians were recruited through local fieldwork partners and were eligible for inclusion if their primary specialty was neurology and they treated 2 or more patients with CIDP in a typical month. Patients were eligible for inclusion if they were aged 18 or over and had a confirmed diagnosis of CIDP.

Neurologists completed a questionnaire for 2 to 10 consecutively consulted patients with a CIDP diagnosis. The questionnaire primarily captured point-in-time data, but also incorporated information from patients’ medical records (treatment history, time from symptom onset to first consultation, time to diagnosis, physician/s involved in initial consultation and diagnosis, misdiagnosis, tests used at diagnosis, disease severity of CIDP at symptom onset). More detailed information on the DSP methodology has been previously described (26, 27), validated (28) and demonstrated to be representative and consistent over time (29). Ethical exemption was obtained from PEARL IRB (protocol #22-ADRW-153).

### Sample

The total sample consisted of 542 patients. Basic demographic data were collected, including age, sex, BMI and country of residence. Neurologists also reported on clinical and diagnostic data. The disease severity was assessed by the physician according to their own definition of the terms mild, moderate and severe. The INCAT disability score, comprised of an arm score and a leg score, was used to determine a patient’s level of impairment at time of survey. The total score can range from 0 (no functional impairment) to 10 (inability to make any purposeful movement) (30).

### Statistical analysis

We assessed the impact of patient characteristics (age category, sex, disease severity at symptom onset, BMI and CIDP subtype) on misdiagnosis and time to diagnosis. The association between two categorical variables was tested for significance using a Chi-squared test. The difference in median time to diagnosis between groups was tested for significance using the Mood’s median test. A multiple linear regression model was fitted to understand the factors affecting the time to diagnosis. A log-transformation was applied to the time to diagnosis to obtain normally distributed residuals. As the patients are clustered within neurologists, a random effect for the neurologist was added to the model. The explanatory power of the model was expressed using the marginal R^2^, being the proportion of variance explained by the fixed effects in the model, the unadjusted intraclass-correlation coefficient, being the proportion of variance explained by the random effect in the model, and the conditional R^2^, being the proportion of variance explained by the fixed effects and random effects in the model (31).

## Results

### Patient characteristics

Table 1 shows patient characteristics, both for the overall sample and by country. Patients’ mean (SD) age was 54.0 (12.4) years. On average, Germany’s patient population was the youngest at 51.9 (10.9) years, while France’s was the oldest at 56.6 (13.8). In the overall sample, 62% of patients were male. At the time of the survey, 85% of patients were receiving treatment for CIDP, with immunoglobulin and corticosteroids being the most frequently administered. The frequency with which these treatments were prescribed varied by country: intravenous immunoglobulin only was administered to 50% of patients in France, compared to only 21% in Germany. Similarly, corticosteroids only were prescribed more often to German patients than French patients (40% vs. 10%). For patients who were not being treated at the time of the survey, the most often cited reason was that their condition was stable without treatment.

**Table 1:** Characteristics of CIDP patients per country and overall.

		Patients					
		Overall (*N* = 542)	France (*N* = 124)	Germany (*N* = 120)	Italy (*N* = 124)	Spain (*N* = 120)	UK (*N* = 54)
Total	Sample size	100%	23%	22%	23%	22%	10%
Sex	Male	62%	65%	56%	63%	64%	65%
Age	Mean (SD)	54.0 (12.4)	56.6 (13.8)	51.9 (10.9)	54.2 (12.4)	53.9 (12.6)	52.4 (10.9)
	18–34	7%	8%	8%	6%	8%	6%
	35–44	14%	10%	15%	17%	9%	20%
	45–54	28%	20%	38%	26%	29%	28%
	55–64	32%	32%	31%	30%	34%	35%
	65–74	14%	19%	8%	15%	17%	11%
	75+	5%	10%	1%	6%	3%	0%
Treatment	Intravenous immunoglobulin	30%	50%	21%	23%	25%	37%
	Subcutaneous immunoglobulin	3%	3%	3%	6%	1%	0%
	Corticosteroids	26%	10%	40%	21%	29%	39%
	Immunoglobulin and corticosteroids	12%	6%	7%	22%	15%	11%
	Other treatment*	13%	16%	21%	9%	10%	7%
	No treatment	15%	15%	7%	19%	20%	6%

		Overall (*N* = 79)	France (*N* = 19)	Germany (*N* = 9)	Italy (*N* = 24)	Spain (*N* = 24)	UK (*N* = 3)
Reasons for not being on treatment	Patient’s condition is stable without treatment	75%	89%	NR	79%	88%	66%
	Patient does not want medication for their CIDP	27%	NR	89%	13%	33%	66%
	Patient is newly diagnosed and will be given treatment at next consultation	6%	11%	11%	8%	NR	NR
	Patient is not eligible for treatment	3%	NR	NR	4%	4%	NR

*Rituximab, azathioprine, tacrolimus, methotrexate, cyclophosphamide, plamapheresis, mycophenolate mofetil. SD, Standard deviation.

In the overall sample, 50% of patients had at least one listed comorbidity, with anxiety, depression and diabetes being the most frequent (Table 2). Depression and anxiety were diagnosed after CIDP in 53% and 46% of the cases, respectively, while diabetes was diagnosed after CIDP in 20% of the cases. Supplementary Table S1 shows the distribution of comorbidities per country: in Germany, the proportion of patients with one or more comorbid conditions was notably lower at 30%. It was also the only country in which physicians did not report anxiety as one of the top two comorbidities. By contrast, 63% of the Spanish sample had one or more comorbidities, with anxiety and depression affecting 24% and 21% of patients, respectively.

**Table 2:** Distribution of comorbidities overall.

	Overall (*N* = 542)	
	% patients with comorbidity	% patients with comorbidity who received diagnosis after CIDP diagnosis
Had one or more comorbid condition(s)	50%	–
Depression	13%	53%
Anxiety	13%	46%
Diabetes	8%	20%
Chronic pulmonary disease	5%	19%
Myocardial infarction	4%	17%
Monoclonal gammopathy (MGUS)	4%	48%
Peripheral vascular disease	4%	23%
Rheumatologic disease	4%	9%
Mild liver disease	4%	21%
Cerebrovascular disease	3%	24%
Peptic ulcer disease	2%	13%
Renal disease	3%	18%
Congestive heart failure	1%	33%
Other	11%	17%

CIDP, Chronic inflammatory demyelinating polyradiculoneuropathy.

### Disease characteristics

The disease characteristics of the overall patient population are shown in Table 3. The majority of patients had been diagnosed with typical CIDP (68%). Among those that had been diagnosed with variant CIDP (32%), multifocal CIDP was the most common. The most common symptoms in the overall sample at time of survey were peripheral numbness (69%), peripheral tingling (64%) and distal muscle weakness (62%). The mean (SD) total INCAT score was 3.1 (1.9). For 74% of patients, physicians judged the severity of their CIDP at diagnosis to be moderate-to-severe. At time of survey, only 49% of patients were considered to have moderate-to-severe CIDP.

**Table 3:** Disease characteristics of CIDP patients overall.

	Patients	
		*N* = 542
CIDP type	Typical CIDP	68%
	Variant CIDP	32%
	Distal CIDP	7%
	Focal CIDP	2%
	Motor CIDP	6%
	Multifocal CIDP	10%
	Sensory CIDP	7%
Disease severity	At diagnosis	*N* = 522
	Mild	26%
	Moderate	53%
	Severe	21%
	At time of survey	*N* = 522
	Mild	51%
	Moderate	43%
	Severe	6%
Symptoms	Peripheral numbness	69%
	Peripheral tingling	64%
	Distal muscle weakness	62%
	Areflexia (loss of deep tendon reflexes)	51%
	Proximal muscle weakness	42%
	Neuropathic pain	36%
	Physical fatigue/low energy	33%
	Difficulty walking/maintaining gait	32%
	Loss of balance/falling	27%
	Peripheral burning	22%
INCAT	Mean (SD) total score	3.1 (1.9)
	Mean (SD) arm score	1.5 (1.1)
	Mean (SD) leg score	1.6 (1.1)

CIDP, Chronic inflammatory demyelinating polyradiculoneuropathy; SD, Standard deviation; INCAT, Inflammatory neuropathy cause and treatment.

### Misdiagnosis and diagnostic pathway

Summary statistics on misdiagnosis in CIDP and the diagnostic pathway are shown in Table 4. For 417 patients, physicians confirmed whether or not they had initially been diagnosed with another condition based on symptoms later attributed to CIDP. In this group, 37% of patients had initially been suspected to have at least one other condition prior to a confirmatory CIDP diagnosis. The top 5 most common misdiagnosed conditions were GBS (37%), fibromyalgia (13%), diabetic polyneuropathy (11%), multiple sclerosis (9%) and toxic neuropathy (7%). Other conditions were reported at a lower frequency and collectively accounted for 29%. For 125 patients, whether another condition had been diagnosed prior to CIDP was unconfirmed.

**Table 4:** CIDP patients’ experience with (mis)diagnosis.

		Proportion of *N* = 417
Number of misdiagnoses	0 (No misdiagnosis)	264 (63%)
	1	139 (33%)
	2	13 (3%)
	3	1 (0%)

		Proportion of *N* = 147
Misdiagnosed conditions (% of misdiagnosed patients)	Guillain-Barré syndrome	37%
	Fibromyalgia	13%
	Diabetic polyneuropathy	11%
	Multiple Sclerosis	9%
	Toxic neuropathy	7%
	Other condition(s)	29%

		*N*	% misdiagnosed
Misdiagnosis by CIDP type	Overall	417	37%
	Typical CIDP	291	35%
	Variant CIDP	126	41%
	Distal CIDP	25	40%
	Focal CIDP	6	33%
	Motor CIDP	26	31%
	Multifocal CIDP	44	45%
	Sensory CIDP	25	48%

Diagnostic pathway	
First consultation	
Time between symptom onset and first consultation (*N* = 373)	
Median (Q1-Q3)	3.0 (1.0–6.4) months
Diagnosis	
Time between first consultation and diagnosis (*N* = 373)	
Median (Q1-Q3)	3.0 (1.0–6.0) months
Time since diagnosis (*N* = 542)	
Median (Q1-Q3)	2.8 (1.1–4.0) years

Diagnosing healthcare professional	
	Proportion of *N* = 540
Neurologist	403 (75%)
Neuromuscular specialist	132 (24%)
Other HCP	3 (1%)
Internist	2 (0%)

CIDP, Chronic inflammatory demyelinating polyradiculoneuropathy; SD, Standard deviation; Q1, First quartile (25% percentile); Q3, Third quartile (75% percentile); HCP, Healthcare professional.

The median (Q1 – Q3) time from symptom onset to the first consultation was 3.0 (1.0–6.4) months, with a maximal value of 267 months. The median (Q1 - Q3) time from the first consultation to diagnosis was 3.0 (1.0–6.0) months, with a maximal value of 155 months. The median (Q1 – Q3) time since diagnosis was 2.8 years (1.1–4.0) at the time of the survey.

The diagnosing healthcare professional (HCP) was most often a neurologist (75%) and sometimes a neuromuscular specialist (24%). A small minority of patients was diagnosed by either an internist or another HCP.

Table 5 shows the 10 most commonly used tests to aid diagnosis of patients with CIDP. The mean (SD) number of diagnostic tests undergone was 19.6 (9.4). An electromyogram and a nerve conduction study (98%) were performed most commonly, followed by a complete blood count (82%).

**Table 5:** Number of diagnostic tests and 10 most used diagnostic tests.

		*N* = 542
Number of diagnostic tests per patient	Mean (SD)	19.6 (9.4)
Tests used to aid diagnosis	Electromyogram and nerve conduction study	98%
	Complete blood count	82%
	Anti-ganglioside antibodies (anti-GM1)	78%
	C-reactive protein	74%
	Anti-myelin associated glycoprotein antibodies	74%
	Antinuclear antibodies	73%
	Antineutrophil cytoplasmic antibodies	71%
	Lumbar puncture/ cerebrospinal fluid testing	70%
	Glycosylated hemoglobin (HbA1c)	68%
	Liver function	68%

SD, Standard deviation.

### Associations between patient/disease characteristics and probability of being misdiagnosed

Table 6 shows the probabilities of being misdiagnosed for patient subgroups defined by sex, disease severity at symptom onset, age category, BMI and CIDP subtype. No statistically significant associations were found.

**Table 6:** Associations between patient characteristics and probability of being misdiagnosed.

		No misdiagnosis (*n*, %)	Misdiagnosis (*n*, %)	*p*-value of Chi-squared test
Sex	Female	102 (66%)	52 (34%)	0.3993
	Male	162 (62%)	101 (38%)	
Disease severity at symptom onset	Mild	106 (65%)	58 (35%)	0.9239
	Moderate	114 (62%)	71 (38%)	
	Severe	36 (65%)	19 (35%)	
Age category	<30	25 (74%)	9 (26%)	0.4082
	30- < 45	52 (60%)	34 (40%)	
	45- < 60	103 (59%)	73 (41%)	
	≥60	34 (58%)	25 (42%)	
BMI	<25	137 (62%)	85 (38%)	0.4710
	25- < 30	116 (66%)	59 (34%)	
	≥30	11 (55%)	9 (45%)	
CIDP subtype	CIDP variant	74 (59%)	52 (41%)	0.2436
	Typical CIDP	190 (65%)	101 (35%)	

BMI, Body mass index; CIDP, Chronic inflammatory demyelinating polyradiculoneuropathy.

### Associations between patient/disease characteristics and the time between symptom onset and diagnosis

The median (Q1-Q3) time between symptom onset and diagnosis was 7.0 (3.2–13.0) months. Table 7 shows the summary statistics of the time between symptom onset and diagnosis for patient subgroups defined by sex, disease severity at symptom onset, age category, BMI, CIDP subtype and one or more previous misdiagnoses. The time between symptom onset and diagnosis was found to be significantly associated with the disease severity at symptom onset, CIDP subtype and prior misdiagnoses. Patients with a longer time to diagnosis more often had mild symptoms at onset, a CIDP variant or prior misdiagnoses.

**Table 7:** Associations between patient characteristics and time between symptom onset and diagnosis.

		*N*	Median (Q1 – Q3) time to diagnosis (months)	*p*-value of test for medians
Sex	Female	158	6.0 (3.2–14.0)	0.276
	Male	273	7.0 (3.6–13.0)	
Disease severity at symptom onset	Mild	155	9.1 (5.0–18.9)	<0.0001
	Moderate	192	5.5 (2.9–11.0)	
	Severe	58	4.0 (2.6–9.7)	
Age category	<30	38	6.3 (4.2–20.5)	0.564
	30- < 45	113	6.9 (3.6–13.1)	
	45- < 60	213	6.7 (2.9–12.1)	
	≥60	67	8.0 (5.0–15.0)	
BMI	<25	224	6.7 (3.7–13.9)	0.309
	25- < 30	184	7.0 (3.0–12.0)	
	≥30	23	11.0 (5.0–19.0)	
CIDP subtype	Variant	131	8.4 (4.1–19.5)	0.004
	Typical	300	6.0 (3.1–11.6)	
Misdiagnosed	No	208	5.0 (2.9–10.0)	<0.0001
	Yes	140	9.1 (4.4–20.0)	

SD, Standard deviation; BMI, Body mass index; CIDP, Chronic inflammatory demyelinating polyradiculoneuropathy.

### Multiple linear regression analysis for the time to diagnosis

We performed a multiple linear regression with the log-transform of the time to diagnosis in months as the outcome, and the disease severity at symptom onset, CIDP type and whether the patient was misdiagnosed (yes or no) as the predictors. All predictors had a significant effect, but the fixed effects in the model had limited explanatory power, with a marginal R^2^ of 7.1%. The unadjusted intraclass-correlation coefficient was 50.1%, meaning that half of the variability in the time to diagnosis observations is explained by the neurologist performing the diagnosis. This brings the conditional R^2^ of the model to 57.2%.

The model estimates are shown in Table 8. Patients with moderate and severe disease severity at symptom onset receive a diagnosis more quickly than patients with mild symptoms, with a reduction factor of 0.74 $\left(=e^{-0.306}\right)$ , and 0.81 $\left(=e^{-0.212}\right)$ , respectively. Additionally, having a CIDP variant increased the time to diagnosis by a factor of 1.22 $\left(=e^{0.199}\right)$ , compared to typical CIDP. Finally, prior misdiagnosis significantly delayed diagnosis, increasing the time by a factor of 1.54 $\left(=e^{0.431}\right)$ .

**Table 8:** Multiple linear regression analysis for the log transform of the time to diagnosis.

	Estimate	95% CI	*t*-value	*p*-value
Intercept	2.065	1.797, 2.334	15.096	<0.0001
Disease severity at symptom onset				
Mild (reference group)	–	–	–	–
Moderate	−0.306	−0.507, −0.106	−2.991	0.003
Severe	−0.212	−0.506, 0.085	−1.413	0.159
CIDP type				
Typical CIDP (reference group)	–	–	–	–
CIDP variant	0.199	0.383, 0.015	−2.115	0.035
Misdiagnosed				
No (reference group)	–	–	–	–
Yes	0.431	0.255, 0.607	4.794	<0.0001

CI, Confidence interval; CIDP, Chronic inflammatory demyelinating polyradiculoneuropathy.

## Discussion

We sought to contribute to the limited evidence on diagnosis of CIDP in the published literature through a description of a large real-world sample of CIDP patients and their experience in in getting diagnosed with CIDP. This study illustrated that CIDP patients’ journey to diagnosis can be lengthy and laborious. Median time from symptom onset to first consultation was 3.0 months (maximal value: 267 months) and median time from first consultation to diagnosis was also 3.0 months, (maximal value: 155 months). It should be noted that the latter maximum value suggests very mild disease, making it difficult to determine if the symptoms for that entire period were indeed related to CIDP. In addition, patients underwent 19.6 diagnostic procedures on average before receiving a confirmatory diagnosis of CIDP. The most frequently used diagnostic tests in our sample correspond to the EAN/PNS guidelines’ strongly advised investigations and investigations to be performed if indicated (2).

The majority of patients in our sample were prescribed treatment at the time of survey, with immunoglobulin and corticosteroids being administered most often. The rates at which these treatments were prescribed differed between countries. One of the reasons for this could be local healthcare systems related to the prescription of immunoglobulin, which can impact clinical practice. As the EAN/PNS provide overarching recommendations regarding treatment but do not mandate a single preferred approach, differences in usage rates naturally occur.

Half of our sample had at least one comorbidity, with anxiety and depression being the top 2 reported comorbidities in almost every country, though proportions varied substantially. The differences between countries may be culture-bound, as a lack of emotional wellbeing may not be considered as a comorbidity in some countries or practices. Concurrent diabetes was reported third most often. A fifth of patients with concurrent diabetes in our sample were diagnosed with diabetes after having been diagnosed with CIDP. However, some may have simply had undiagnosed diabetes prior to the onset of CIDP. The rate of diabetes in our cohort was lower than in recent literature from the UK, Serbia and Italy (32, 33). These studies suggest a two-fold risk of diabetes in subjects with CIDP, which is supported by reported rates in cohorts and epidemiological studies from other countries (34, 35). Compared to the study by Doneddu et al. (32), the mean age and the proportion of patients having at least one comorbidity were lower in our cohort (54 years old and 50% vs. 58 years old and 75% in theirs, respectively), which may explain the lower rate of diabetes.

Over a third of patients in our sample were misdiagnosed at least once, though some of these misdiagnoses could represent an initial attempt by a general healthcare practitioner rather than a specialist. GBS represented over a third of misdiagnoses. It can be debated if GBS is a true misdiagnosis, as acute-onset CIDP presents in a similar way to GBS and the diagnosis only becomes apparent with time, demonstrating continuing progression or successive relapses. No statistically significant associations were observed between patient characteristics (age category, sex, disease severity at onset, BMI and CIDP subtype) and the likelihood of misdiagnosis. However, regression analysis indicated that patients who presented with mild symptoms at onset, had variant CIDP or had initially been misdiagnosed on average took longer to receive a diagnosis of CIDP. Previous literature has highlighted that CIDP variants are more challenging to diagnose and have a higher probability of initial misdiagnosis (4, 12, 14, 16, 22). Our findings are consistent with this, with patients that suffered from variant CIDP having a longer time to diagnosis. However, while the proportion of misdiagnoses was higher in patients with variant CIDP (41%) than typical CIDP (35%), this difference was not statistically significant.

The consequences of delayed diagnosis are multiple. In the short-term, patients are delayed in receiving symptom-alleviating immunomodulatory treatment. In the long-term, their physical and mental health, as well as their quality of life, are likely to suffer major consequences, with patients potentially sustaining permanent nerve damage if they are not treated. A recent multicenter study of 144 subjects from Korea and the UK demonstrated poorer outcomes when treatment was commenced over a year after symptom onset as compared to within a year (36). These findings are in keeping with the occurrence of irreversible axonal loss with delayed diagnosis and treatment.

Our study has a number of limitations: first and foremost, it solely utilized physician-reported data, preventing us from capturing the patient’s perspective. Patients included in the DSP sample may not be truly representative of the overall population of CIDP patients, as patients who consult with health care providers more frequently are more likely to be included. In addition, only patients aged 18 and older were included, preventing the capture of diagnostic data for minors with CIDP, a demographic for which little real-world data has been collected thus far. The quality of the data is also dependent on the reporting accuracy of information by physicians which may be subject to recall bias. Additionally, information on misdiagnosis was not available for all patients. Furthermore, this survey captured point-in-time data which, by its very nature, is unable to capture the complete burden associated with a progressive and relapsing/remitting disease such as CIDP. Finally, according to the European Academy of Neurology and Peripheral Nerve Society guidelines, autoimmune nodopathies should not be classified as CIDP, because they show a different clinical manifestation and respond poorly to intravenous immunoglobulin (2). Different studies have shown that Rituximab is effective in patients with an autoimmune nodopathy (37, 38). Nevertheless, nodal and antinodal antibodies can be found in 10–15% of patients diagnosed with CIDP (16). No distinction was made between CIDP and autoimmune nodopathies in the DSP. Despite these limitations, real-world data on CIDP patients’ diagnostic experience is valuable due to its scarcity, especially for large samples. To our knowledge, this is the largest real-world study describing this aspect of the disorder to date.

From our analysis, we conclude that the journey to diagnosis for patients suffering from CIDP is a difficult one: median time to diagnosis in our sample was 7 months and a third of patients were at some point misdiagnosed. The heterogeneous presentation of CIDP, including its variant forms, further complicates timely diagnosis. Mild symptoms at onset, having variant CIDP and having been misdiagnosed were found to be associated with a longer time to diagnosis. In view of recently reported poorer outcomes with delayed treatment, there is a clear need for optimization of the diagnostic process, and for further research into the factors that contribute to delays.

## Data Availability

The data analyzed in this study is subject to the following licenses/restrictions: Not publicly available, but upon reasonable request and with permission of Adelphi Real World, access to the dataset can be granted. Requests to access these datasets should be directed to yasmin.taylor@omc.com.
