# Supplementary material for: The journey to diagnosis for patients with CIDP: results from a real-world international survey
Source: Front Neurol. 2026 Feb 4;16:1748903. doi: 10.3389/fneur.2025.1748903 (PMC12913127; doi:10.3389/fneur.2025.1748903)
Supplement: Supplementary file 1 [file Table_1.DOCX]

**Table S1. Distribution of comorbidities per country**

|  | **% patients with comorbidity per country** | | | | |
| --- | --- | --- | --- | --- | --- |
|  | **France (N=124)** | **Germany (N=120)** | **Italy (N=124)** | **Spain (N=120)** | **UK (N=54)** |
| **Had one or more comorbid condition(s)** | **56%** | **30%** | **52%** | **63%** | **50%** |
| Depression | 13% | 8% | 15% | 24% | 11% |
| Anxiety | 12% | 4% | 14% | 21% | 11% |
| Diabetes | 9% | 3% | 13% | 7% | 9% |
| Chronic pulmonary disease | 7% | 3% | 6% | 7% | 9% |
| Myocardial infarction | 6% | 3% | 6% | 7% | 7% |
| Monoclonal gammopathy (MGUS) | 5% | 2% | 5% | 6% | 6% |
| Peripheral vascular disease | 4% | 2% | 5% | 6% | 4% |
| Rheumatologic disease | 3% | 2% | 4% | 5% | 4% |
| Mild liver disease | 3% | 2% | 3% | 3% | 2% |
| Cerebrovascular disease | 2% | 2% | 3% | 3% | 2% |
| Peptic Ulcer Disease | 2% | 1% | 2% | 2% | 2% |
| Renal disease | 0% | 0% | 2% | 2% | 2% |
| Congestive heart failure | 0% | 0% | 2% | 2% | 0% |
| Other | 14% | 5% | 13% | 14% | 6% |
